# The Sufferings of Christ

**Published:** 1888-02-04

**Authors:** 


### Words of Consolation.

[Communications for this column should be addressed "Chaplain," care of the Editor of The Hospital, who will gratefully welcome any suggestions or inquiries from matrons, nurses, and the staff generally.]

### LXV.—The Sufferings of Christ.

*For Reading to the Sick.*

We have seen, so far, how our blessed Lord bore the sins of many in His own body, and carried them indeed with Him on to the tree of shame. When the cross was raised He had endured in detail the consequences of almost every separate sin we can think of—envy, cruelty, hatred, spite, hard words, dishonour, shame, injustice, false accusation, meanness, cursing. And all this was in one sense the result of the covetousness of one man. If you want to know exactly how Jesus bore your sins we cannot lead you into depths of the sufferings which may for ever remain a mystery. Nevertheless, we may reverently suggest that by looking at the Passion prayerfully and steadily you will find almost any particular sin you may have been guilty of inflicted upon Him, in so far as that He endured consequences due to it. It would be hard to find any sin which was not at some period of the Passion "made His," in the sense of His bearing just the pain which belongs to that sin when inflicted by one person upon another. This we say with regard to all sin in so far as it is not only disobedience in itself, but an injury to God's creatures. If satisfaction for secret sins does not seem to be visible in the Atonement, we shall at least find some of their dire consequences represented in the anguish of the last agony in the darkness. Once more, What is the use of taking to heart so many details of the Passion? Why are we given such minute accounts of our Lord's last hours, of all the night before, of the morning, as well as of the Crucifixion? Why; but that we may each make sure of crucifying the member or members of our own bodies which we know to have been the worst offenders, and then proceed to mortify the affections and lusts from which the corruption sprang. So being crucified and buried with our Saviour here, we shall pass on safely through the grave and gate of death to a joyful resurrection. Otherwise it is evident from the light of the New Testament that any who continue in sin, at length place themselves outside the range of the atonement, seeing that they crucify the Son of God afresh, and put Him to an open shame. It is not less plain that unrenewed nature, by reason of the terrible forces at work within itself, must inevitably, if left to go its own way, bring to a terrible perfection the retribution already commenced in the flesh. "Be not deceived; God is not mocked. Whatsoever a man soweth that shall he also reap. For he that soweth to his flesh shall of the flesh reap corruption; but he that soweth to the Spirit shall of the Spirit reap life everlasting." (Gal. vi. 7, 8.) Those who believe in a law of retribution will not be content with any faith in Christ crucified which does not promise them deliverance from the power as well as from the guilt of sin. To be forgiven, and next to realize forgiveness, is a blessing indeed. But alas! it is awful to think how many Christians base their hopes for eternity upon the bare cancelling of guilt, forgetting that remission of sins can be of little avail so long as the affections and desires are not raised, and so far pointed in another direction as to show that the dominion of sin has ceased.

The more I see of our national faults and miseries the more they resolve themselves into conditions of childish illiterateness and want of education in the most ordinary habits of thought.—*Ruskin.*

